# Limited copy number - high resolution melting (LCN-HRM) enables the detection and identification by sequencing of low level mutations in cancer biopsies

**DOI:** 10.1186/1476-4598-8-82

**Published:** 2009-10-08

**Authors:** Hongdo Do, Alexander Dobrovic

**Affiliations:** 1 Department of Pathology, University of Melbourne, Parkville, Victoria, 3010, Australia; 2 Molecular Pathology Research and Development Laboratory, Department of Pathology, Peter MacCallum Cancer Centre, Locked Bag 1, A'Beckett St, Melbourne, Victoria 8006, Australia

## Abstract

**Background:**

Mutation detection in clinical tumour samples is challenging when the proportion of tumour cells, and thus mutant alleles, is low. The limited sensitivity of conventional sequencing necessitates the adoption of more sensitive approaches. High resolution melting (HRM) is more sensitive than sequencing but identification of the mutation is desirable, particularly when it is important to discriminate false positives due to PCR errors or template degradation from true mutations.

We thus developed limited copy number - high resolution melting (LCN-HRM) which applies limiting dilution to HRM. Multiple replicate reactions with a limited number of target sequences per reaction allow low level mutations to be detected. The dilutions used (based on Ct values) are chosen such that mutations, if present, can be detected by the direct sequencing of amplicons with aberrant melting patterns.

**Results:**

Using cell lines heterozygous for mutations, we found that the mutations were not readily detected when they comprised 10% of total alleles (20% tumour cells) by sequencing, whereas they were readily detectable at 5% total alleles by standard HRM. LCN-HRM allowed these mutations to be identified by direct sequencing of those positive reactions.

LCN-HRM was then used to review formalin-fixed paraffin-embedded (FFPE) clinical samples showing discordant findings between sequencing and HRM for *KRAS *exon 2 and *EGFR *exons 19 and 21. Both true mutations present at low levels and sequence changes due to artefacts were detected by LCN-HRM. The use of high fidelity polymerases showed that the majority of the artefacts were derived from the damaged template rather than replication errors during amplification.

**Conclusion:**

LCN-HRM bridges the sensitivity gap between HRM and sequencing and is effective in distinguishing between artefacts and true mutations.

## Background

The recent demonstrations of the clinical benefits of molecularly targeted therapies [1,2] have greatly increased the demand for cost-effective and reliable methods of mutation detection in individual tumour samples. However, clinical tumour samples are typically heterogeneous, containing a mixture of cancer cells and various normal cell types. The proportion of cancer cells can be quite low and often limited amounts of material are available for analysis. The cancer cells may also show clonal heterogeneity, and thus a particular genetic change may not be present in all of the tumour cells [3]. All of these factors present challenges for mutation detection in clinical samples.

Dideoxynucleotide sequencing is often considered the gold standard for detection of genetic changes as it allows these changes to be directly characterised. However, to be reliably detected, changes are required to be present at a proportion of at least 10-20% at the allelic level [4]. Thus, lower levels of mutations, due to high contamination with normal cells or tumour heterogeneity, can not be detected by sequencing when the mutations are present below the sensitivity of sequencing.

Various PCR-based techniques for detecting sequence variation have been reported, demonstrating both advantages and disadvantages in terms of sensitivity, simplicity, cost-efficiency and robustness. This article will focus on the detection of *EGFR *and *KRAS *mutations, although the principles can be extended to mutations in any gene. Reported techniques available for detection of mutations in these genes include; dideoxynucleotide sequencing, single strand conformation analysis [5], TaqMan PCR [6], amplification refractory mutation system monitored by Scorpion probes [7], denaturing high pressure liquid chromatography [8], pyrosequencing [9], massively parallel sequencing [10,11], cold-PCR [12], mutant-enriched PCR [13] and high resolution melting [14,15].

High resolution melting (HRM) is an in-tube scanning methodology that detects sequence variation by monitoring the melting behaviour of PCR amplicons using DNA intercalating fluorescent dyes. HRM has been used for a variety of genetic and epigenetic studies [14-18]. It has been demonstrated to be more sensitive than dideoxynucleotide sequencing for the detection of somatic mutations [19]. In our previous study, *KRAS *mutations were able to be detected at levels as low as 5% by HRM [14].

Like other scanning methods, HRM results often need to be confirmed and characterised by a sequencing methodology. Dideoxynucleotide sequencing is the most commonly used methodology. Discordant results between sequencing and HRM may be anticipated if the mutant alleles are present at 5 to 20% due to the different sensitivity of the two methods.

In this study, we report a new method, LCN-HRM, for detection and subsequent identification by sequencing of low levels of sequence variation, particularly those that are only detectable by HRM. The basic principle of this methodology is that low level mutant alleles which cannot be detected in the presence of large amounts of normal alleles are present at higher proportions when the sample is diluted at low copy numbers in multiple aliquots (limiting dilution) [20,21]. The range of copy numbers are chosen so that aliquots containing mutations can be detected with HRM and the mutations can then be characterised by direct sequencing of the aberrant amplicons.

## Methods

### Samples and DNA extraction

This study was covered by an approval from the Peter MacCallum Cancer Centre Ethics Committee (project number 03/90). Two cancer cell lines, HCT116 and NCI-H1650, were used. HCT116 contains a *KRAS *exon 2 missense mutation whereas NCI-H1650 harbours a *EGFR *exon 19 deletion mutation. Peripheral blood from four normal individuals was used as a source of DNA for the quantitative estimation of target copy number and as normal DNA for the cell line dilution experiments. Genomic DNA was extracted from the cell lines and peripheral blood using the QIAamp DNA blood kit (Qiagen, Hilden, Germany) according to the manufacturer's instructions. DNA was quantified with a NanoDrop ND-1000 Fluorospectrometer (NanoDrop, Wilmington, DE). Four formalin-fixed paraffin-embedded (FFPE) DNA samples from non-small cell lung cancer (NSCLC) patients were included to investigate the cause of previously reported discrepancies between HRM and sequencing results [15].

### PCR and HRM conditions

Real time PCR amplification and HRM were performed on the Rotor-Gene Q (Qiagen, Hilden, Germany). The HRM primers for *KRAS *exon 2 [14] and *EGFR *exon 19 [15] generated 189 bp and 250 bp products respectively. The conditions for *KRAS *exon 2 were as follows. The reaction mixture (20 μl final volume) contained the template, 1× PCR buffer, 2.5 mM MgCl_2_, 200 nM each primer, 200 μM dNTPs, 5 μM SYTO 9 (Invitrogen, Carlsbad, CA) and 0.5 U HotStarTaq (Qiagen). The cycling and melting conditions were; one cycle of 95°C for 15 minutes; 55 cycles of 95°C for 10 seconds, 67.5°C for 5 seconds with an initial 10 cycles of touchdown from 67.5°C to 57.5°C (1°C/cycle), 72°C for 20 seconds; one cycle of 97°C for one minute and a melt from 70°C to 95°C rising 0.2°C per second. For LCN-HRM, the conditions were the same except that 60 cycles of amplification were used. The cycling conditions for the *EGFR *exon 19 assay were identical except that the initial annealing temperature was 65°C. The melting profiles of amplicons were analysed using the Rotor-Gene Software (v1.7).

### Preparation of standard dilutions using quantitative real-time PCR (qPCR)

The amount of amplifiable template for each DNA sample is most accurately estimated by qPCR, particularly when an accurate dilution series is required. For high quality DNA, any amplicon should yield similar results. We used previously reported *KRAS *primers which generated a 189 bp product [14] and *EGFR *primers which generated a 250 bp product [14]. PCR conditions were as above. The cycle threshold (Ct) value was determined by setting the threshold at 0.1 in the quantitation analysis of the Rotor-Gene software. Using the qPCR data, a range of cell line DNA dilutions from 20% to 1% mutant allele frequency was prepared.

### qPCR for copy number estimation prior to limiting dilution

When dealing with FFPE specimens, the degree of fragmentation will affect the amount of template molecules that can be amplified. The effective copy number of amplifiable template for each DNA sample is again most accurately estimated by qPCR. In high quality DNA, there is an average of one copy of an autosomal gene per 3 pg of DNA. The effective copy number in the sample is obtained by comparing the Ct of the sample with the Ct value of a high quality reference. To correct for the variable amounts of DNA fragmentation, the amplicon used for the estimation needs to be in the same size range as the amplicons being screened for mutations. It is ideal to use the same amplicon that is being screened.

The qPCR data was used for calculation of the dilution factor which gave approximately four copies of template (12 pg) per reaction. To estimate the necessary dilution, the Ct value of the sample (*Ct*_*s*_) was compared to the Ct value of reference DNA (*Ct*_*ref*_) of a known concentration. The comparative quantitation analysis of the Rotor-Gene software was used to calculate the PCR efficiency (E) and the relative amounts of amplifiable template are related the formula E^(*Ctref *- *Cts*)^. The amounts of template of each test sample were then accordingly diluted.

### Sensitivity determination of sequencing and HRM

Dilutions of the *KRAS *mutant HCT116 cell line (20%, 10%, 5% and 1%) were used to assess the relative sensitivity of DNA sequencing and HRM. The sensitivity testing was performed with primers and conditions for the 189 bp *KRAS *product as above. Sequencing reactions for each DNA dilution were performed using the Big Dye Terminator v3.1 (Applied Biosystems, Foster City, CA) and the results were analysed using Sequencher 4.6 (Gene Codes Corporation, Ann Arbor, MI).

### Detection of low levels of mutation by LCN-HRM in reconstruction experiments

The *KRAS *and *EGFR *mutant cell lines, HCT116 and NCI-H1650, were used to show that LCN-HRM can detect low levels of different types of mutations (missense mutations and deletions respectively) and also to show that the mutations can be characterised by subsequent sequencing of individual LCN-HRM positive reactions.

Each cell line DNA was initially diluted at 5% mutant allele frequency by mixing with normal peripheral blood DNA based on qPCR data. The 5% cell line DNA mixtures were then further diluted to an estimate of average four copies in a one microlitre volume. Both cell line DNA dilutions were tested by LCN-HRM in 65 replicates with known positive and negative controls.

### Quantitation of low-levels of mutant alleles by LCN-HRM

The frequency of a mutant allele may be estimated by analysis of LCN-HRM reactions. The frequency of LCN-HRM positive reactions approximates the amount of mutant alleles present in the sample when the mutation frequency is low.



For example, if 25 out of 72 LCN-HRM reactions (which were tested with an average of four copies per reaction) were scored as positive by melting curve analysis, the estimated frequency of the mutant allele would be 8.7% {25/(72 × 4)}. As the frequency of the mutant allele increases or the average copy number per reaction increases, this formula will become less accurate due to the possibility of two mutant sequences being found in one reaction.

### LCN-HRM of FFPE samples

DNA was previously extracted from four selected NSCLC samples (TX13, TX49, TX86 and TX450) [15]. The first three samples were positive for *EGFR *mutations by HRM but not sequencing and the last sample was positive for *KRAS *mutation by HRM but not sequencing. We tested those samples by LCN-HRM to review the discrepancies between HRM and the sequencing results. *KRAS *exon 2 LCN-HRM conditions were as above. TX13 was tested for mutations in *EGFR *exon 19 (250 bp product), and TX49 and TX86 were screened for mutations in *EGFR *exon 21 (212 bp product). *EGFR *LCN-HRM assays were carried out as previously described for HRM assays [14] except that 65 cycles of amplification were performed. All samples were tested in 64 replicates using an estimated three to four templates per LCN-HRM reaction except TX13 which was tested with less template (estimated 1 template per reaction) due to the limited template available.

Positive reactions showing aberrant melting profiles were selected and individually sequenced to verify the LCN-HRM results. The selected LCN-HRM products were directly column-purified using the PCR-M clean up kit (Viogene, Taipei, Taiwan) and were eluted in a final volume of 30 μl.

### LCN-HRM using DNA polymerases with proofreading activity

Two different reagent kits with proofreading activity were assessed for their ability to accurately detect mutations: the FastStart High Fidelity PCR System (Roche) and HotStar HiFidelity polymerase (Qiagen). The fidelity was reported to be approximately four-fold (FastStart High Fidelity System) and ten-fold (HotStar HiFidelity) higher than HotStarTaq DNA polymerase (Qiagen). TX84 DNA was tested using LCN-HRM for *EGFR *exon 21 using the two kits. LCN-HRM was performed on the Rotor-Gene Q for both.

For the FastStart High Fidelity PCR System (Roche), the reaction mixture in a final volume of 20 μl contained; 1× PCR buffer, 2.5 mM MgCl_2_, 200 nM each primer, 200 μM dNTPs, 5 μM SYTO 9, 1 U FastStart High Fidelity polymerase. The cycling conditions were; one cycle of 95°C for 2 minutes; 60 cycles of 95°C for 10 seconds, 65°C for 10 seconds with an initial 10 cycles of touchdown (1°C/cycle), 72°C for 30 seconds; one cycle of 97°C for one minute and a melt from 70°C to 95°C rising 0.2°C per second.

For HotStar HiFidelity enzyme (Qiagen), the reaction mixture in a final volume of 20 μl contained; 1× PCR buffer, 1.5 mM MgSO_4_, 200 nM each primer, 1.5 mM dNTPs, 5 μM SYTO 9, 0.5 U HotStar HiFidelity polymerase. The cycling conditions were; one cycle of 95°C for 5 minutes; 60 cycles of 95°C for 10 seconds, 67.5°C for 10 seconds with an initial 10 cycles of touchdown (1°C/cycle), 72°C for 60 seconds; one cycle of 97°C for one minute and a melt from 70°C to 95°C rising 0.2°C per second. All conditions were tested in 64 replicates.

## Results

### The sensitivity of mutation detection by sequencing and HRM

The relative sensitivity of mutation detection by dideoxynucleotide sequencing and HRM was compared using dilutions of HCT116 DNA (heterozygous for the c.38G>A *KRAS *mutation) in normal DNA. To ensure the maximum accuracy in the sensitivity determination, qPCR of the *KRAS *amplicon was used to estimate the effective concentration of each DNA sample. The HCT116 cell line DNA was then serially diluted into normal DNA at ratios of 40%, 20%, 10% and 2% to give mutant allele frequencies of 20%, 10%, 5% and 1%.

**Figure 1:**
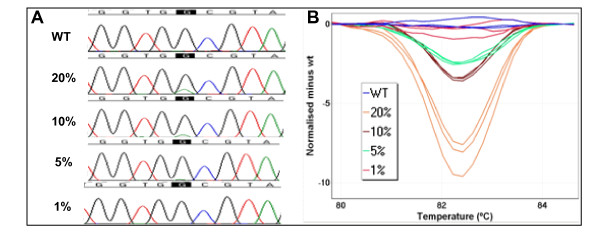
**Comparison of the mutation detection sensitivity of high resolution melting (HRM) and sequencing**. The sensitivity of *KRAS *HRM and dideoxynucleotide sequencing was tested using four HCT116 DNA dilutions. Based on qPCR data, the HCT116 DNA was mixed with wild-type DNA to dilute the mutant allele to 20%, 10%, 5%, and 1% of the total alleles. Sequencing was only sensitive to 10-20% whereas HRM readily detected 5% mutant sequence. Panel A: Sequencing traces of four HCT116 DNA dilutions. The mutant A allele was readily detectable at a 20% mutant frequency. However, when the mutant allele was present at 10%, it was barely distinguishable from the sequencing background. When the frequency of the mutant was below 10%, the mutation was not detectable by dideoxynucleotide sequencing. Panel B: Using HRM, the mutation was readily detectable down at 5% mutation frequency. The 1% dilution was not distinct from the normal DNA. The melting curves of each dilution are shown in orange (20%), brown (10%), green (5%), red (1%) and blue (wild-type control).

Dideoxynucleotide sequencing and HRM were performed at each of the mutant allele frequencies (Figure 1). The mutant A allele was still detectable (at a low peak height) when the mutant allele frequency was 20%. However, when the mutant allele was present at 10%, it was only distinguishable from the sequencing background because the site of mutation was known. When the frequency of the mutant was below 10%, the mutation was not detectable by dideoxynucleotide sequencing. Using HRM, the 5% dilution was readily detectable. The 1% dilution was not distinguishable from the normal DNA controls.

### Detection of low levels of mutation by LCN-HRM

**Figure 2:**
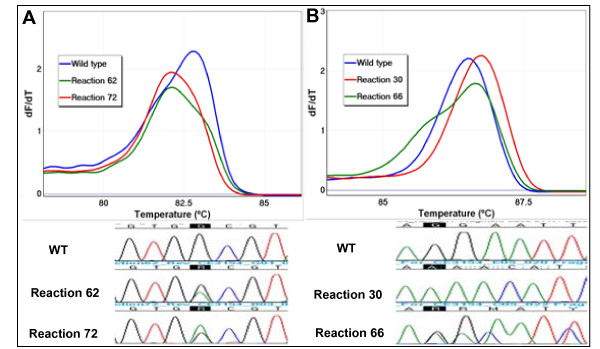
**Detection of low levels of mutations by LCN-HRM and characterisation by subsequent sequencing**. HCT116 and NCI-H1650 cell line DNA were mixed with normal DNA to a 5% mutant allele frequency based on the previous qPCR data. Each of the DNA mixtures were then tested by LCN-HRM in 65 replicates. An estimated average of three copies per reaction were added into the individual reaction. LCN-HRM using the diluted 5% HCT116 and 5% NCI-H1650 was performed for *KRAS *exon 2 and *EGFR *exon 19 respectively. LCN-HRM positive reactions, which were detected by melting curve analysis, were directly sequenced. The first derivative melting plots and sequencing traces of two of the representative LCN-HRM positive reactions are shown. (Panel A for 5% HCT116 and Panel B for 5% NCI-H1650). The identical *KRAS *mutation to the HCT116, c.38G>A, was detected by sequencing of reactions 62 and 72, which displayed aberrant melting pattern compared with wild type. The *EGFR *delE746_A750 was detected heterozygously in reaction 66 and a homozygously in reaction 30 by sequencing.

HCT116 and NCI-H1650 DNA dilutions with a 5% mutant allele frequency were sequencing negative for *KRAS *exon 2 missense and *EGFR *exon 19 deletion mutations respectively (Figure 1, data not shown for *EGFR *exon 19). The same DNA mixtures were tested by LCN-HRM with an estimate of average of three copies of template per reaction. Melting curve analysis of LCN-HRM amplicons led to the identification of positive reactions which were expected to contain mutant alleles. 8/58 LCN-HRM reactions of the 5% HCT116 DNA mixture and 12/59 reactions of the 5% NCI-H1650 DNA mixture showed aberrant melting. Subsequent sequencing of those positive reactions clearly showed the presence of the c.38G>A *KRAS *mutation and delE746_A750 *EGFR *mutation respectively (Figure 2).

### The possible causes of discrepant results between sequencing and HRM

**Figure 3:**
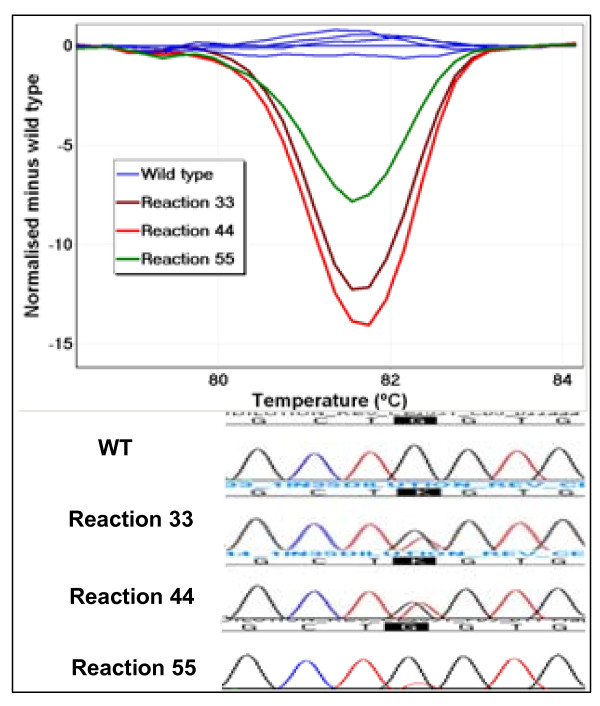
**Detection of a *KRAS *mutations by LCN-HRM in a sequencing negative clinical sample**. Testing of a clinical non-small cell lung cancer sample, TX450, for *KRAS *exon 2 by sequencing gave a negative result whereas the sample was positive by HRM. LCN-HRM was thus performed in 64 replicates using an estimate of average four copies per reaction. The difference graph plot and sequencing traces of three representatives of LCN-HRM positive reactions are shown. A *KRAS *c.34G>T was detected in all three reactions by subsequent sequencing.

Four NSCLC samples extracted from FFPE tissues were tested by LCN-HRM to investigate the cause of discrepant results between HRM and sequencing. TX450 was negative by sequencing but positive by HRM for *KRAS *exon 2 [15]. After LCN-HRM which was performed with estimated averages of four and seven copies per reaction, the melting pattern of each individual reaction was analysed. A total of 18/64 and 30/64 LCN-HRM reactions, displaying heteroduplex melting patterns or a shift of T_m _were scored as positive with four and seven copies respectively. The frequency of mutant alleles was estimated to be 7% with four copies {18 LCN-HRM positives/(64 × 4)} and 6.7% with seven copies {30 LCN-HRM positive/(64 × 7)}. The same *KRAS *mutation, c.34G>T (p.G12C), was repeatedly detected when individual LCN-HRM positive reactions (9 from the reactions with four copies and 10 from the reactions with seven copies) were sequenced (Figure 3). Even though, the mutant allele was detectable in reactions with both four and seven copies, it was predictably more readily detectable with four copies, resulting in reliable characterisation of mutation by sequencing of multiple LCN-HRM positive reactions.

Non-identical *KRAS *sequence variants were also detected in five of the other LCN-HRM reactions; c.22G>A, c.32C>A, c.38G>A, c.59C>T and c.100C>T. These transitional changes have not been previously reported, except those at positions 32 and 38. A transitional C>T change, as opposed to a tranversional C>A, has been reported at c.32 in a few cases whereas G>A change at c.38 (codon 13) has been frequently reported. This illustrates the potential dangers of working from just one sequence in a digital or low copy number situation.

**Figure 4:**
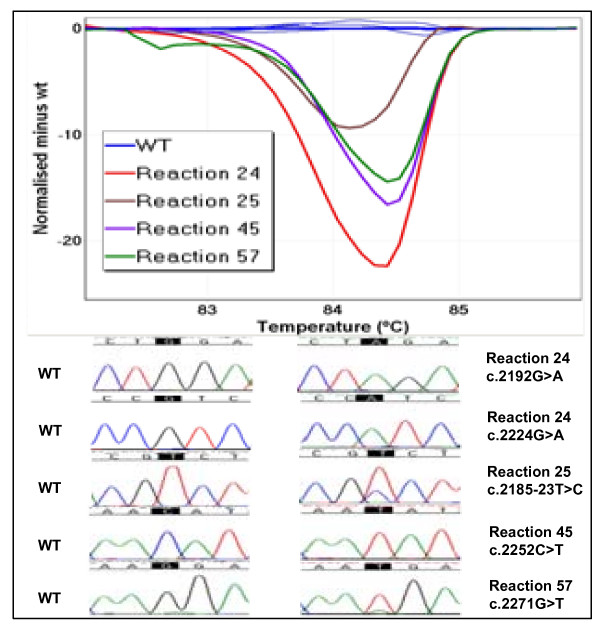
**PCR artefacts detected in in a clinical FFPE sample (TX13)**. A total of 8 out of 31 LCN-HRM reactions were positive by melting curve analysis. Five non-identical sequence variants were detected from 4 of the 8 LCN-HRM positive reactions sequenced. In reaction 24, two transitional G>A changes were detected at positions c.2192 and c.2224. Two other exonic (c.2252C>T and c.2271G>T) and one intronic (c.2185-23T>C) variants were also detected in reactions 45, 57 and 25 respectively.

Three other FFPE samples from NSCLC patients were also investigated by LCN-HRM for mutations in *EGFR *exons 19 (TX13) and 21 (TX49, TX86). After sequencing the LCM-HRM positive products, multiple non-identical sequence variants at different positions were detected in all three samples. Those sequence variants are highly likely to be artefacts. A total of 5 different *EGFR *sequence variants, four exonic and one intronic, were found in TX13 (Figure 4). All of the variants were single base substitutions with predominantly transitional changes (4/5). Among these, two different sequence variations (c.2192G>A and c.2224G>A) were found in one amplicon (reaction 24). Similarly, a total of 12 and 7 sequence variants, again predominantly transitional changes, were identified in TX49 (Table 1) and TX86 respectively (Table 2). Interestingly, a c.2488G>A change was found in two independent amplicons of TX49. This mutation has not been previously reported. This is difficult to interpret as it may either represent a low level mutation in a background of sequencing artefacts or a high probability sequencing artefact.

**Table 1:** Sequence variants detected in a NSCLC FFPE sample, TX49, by LCN-HRM.

**Sample**	**Position**	**Base change**	**Amino acid change**	**Reported**
TX49	c.2488	G>A	p.D830N	No
	c.2488	G>A	p.D830N	No
	c.2499	G>A	p.L833L	No
	c.2502	G>A	p.V834V	No
	c.2506	C>T	p.R836C	Yes
	c.2507	G>T	p.R836L	No
	c.2519	C>T	p.A840V	No
	c.2526	C>T	p.N842N	No
	c.2577	C>T	p.A859A	No
	c.2581	C>T	p.L861L	No
	c.2593	G>A	p.E865K	Yes
	c.2603	A>G	p.E868G	Yes
	c.2625+1	G>A	-	No

**Table 2:** The sequence variants detected in TX86 by LCN-HRM using three different DNA polymerases.

**Polymerase**	**Position**	**Base change**	**Amino acid change**	**Reported**
HotStarTaq (Qiagen)	c.2515	G>T	p.A839S	No
	c.2519	C>T	p.A840V	No
	c.2527	G>A	p.V842I	Yes
	c.2532	G>A	p.L844L	No
	c.2583	G>A	p.L861L	No
	c.2588	G>A	p.G863D	Yes
	c.2592	G>A	p.A864A	No

FastStart High Fidelity (Roche)	c.2602	G>A	p.E868K	No
	c.2494	C>T	p.R832C	No
	c.2512	C>T	p.L838L	No
	c.2570	G>T	p.G857V	No
	c.2576	C>T	p.A859V	No

HotStar HiFidelity (Qiagen)	c.2478	C>A	p.N826K	No
	c.2484	G>T	p.L828F	No
	c.2488	G>T	p.D830Y	No
	c.2492	G>A	p.R831H	Yes
	c.2494	C>T	p.R832C	No
	c.2508	C>A	p.R836R	SNP
	c.2508_2509	CG>TA	p.R836R, p.D837N	No
	c.2509	G>T	p.D837Y	No
	c.2515	G>A	p.A839T	Yes
	c.2517	A>G	p.A839A	No
	c.2522	G>T	p.R841M	No
	c.2530	C>A	p.L844M	No
	c.2543	C>A	p.P848Q	No
	c.2581	C>A	p.L861M	No
	c.2591	C>T	p.A864V	No
	c.2607	C>A	p.Y869X	No
	c.2622	C>A	p.G874G	No

### LCN-HRM using DNA polymerases with proofreading activity

As the LCN-HRM assays were carried out with a *Taq *DNA polymerase lacking proofreading activity, we investigated whether the PCR artefacts were caused by polymerase error or chemical damage to the FFPE DNA by using two DNA polymerases having different levels of fidelity. We hypothesised that if PCR artefacts were entirely DNA polymerase mediated, the frequency of PCR artefacts would decrease as the polymerase fidelity increased. The fidelity of the polymerases were reported to be approximately four-fold (FastStart High Fidelity System, Roche) and ten-fold (HotStar HiFidelity, Qiagen) higher compared to the *Taq *polymerase.

One DNA sample (TX86) was tested with all three polymerases. When the *Taq *polymerase lacking proofreading activity (HotStar *Taq*) was used, nine out of 58 reactions (15.5%) were positive by LCN-HRM. A similar LCN-HRM positive rate was detected with the FastStart High Fidelity enzyme. Five out of 32 reactions (15.6%) were positive. Surprisingly, the highest LCN-HRM positive rate was found with the HotStar HiFidelity enzyme. Twenty-eight of 53 (52.8%) reactions were positive by LCN-HRM (Table 3).

**Table 3:** The frequency of LCN-HRM positives measured with different DNA polymerases in a NSCLC FFPE sample, TX86.

**LCN-HRM**	**HotStarTaq** **(Qiagen)**	**FastStart High Fidelity** **System (Roche)**	**HotStar HiFidelity** **Polymerase (Qiagen)**
Proofreading*	0	4-fold	10-fold
Positive	9 (15.5%)	5 (15.6%)	28 (52.8%)
Negative	49 (84.5%)	27 (84.4%)	25 (47.2%)
Total	58 (100%)	32 (100%)	53 (100%)

* Proofreading activity was relatively compared to that of a *Taq *DNA polymerase. HRM: high resolution melting.

Numerous non-identical sequence variants were detected in the sequencing of those 42 LCN-HRM positive reactions (Table 2). The sequence variants were likely to be PCR artefacts and the frequency of PCR artefacts was not reduced with DNA polymerases having different levels of proofreading activity, indicating that the artefacts were principally due to damaged template.

## Discussion

### Overcoming the limits of detection of sequencing by LCN-HRM

Molecular diagnostic testing of clinical tumour samples, in which the tumour cells may comprise a small part of the cellular material and where the quantity of sample for analysis is limited, necessitates the use of highly sensitive methods which are performable with low levels of template. The gold standard method has been sequencing but newer more sensitive methods often give results which cannot be validated by the less sensitive conventional sequencing methodology.

We developed a new screening method, LCN-HRM, for the sensitive detection of sequence variation. LCN-HRM uses limiting dilution of the DNA template for stochastic mutant allele enrichment [20] followed by HRM for rapid screening. The dilutions used are chosen such that mutations if present can be detected well within the analytic sensitivity of both HRM and conventional sequencing. Thus any sequence variant present in aberrant melts can be readily identified as the HRM products may be directly sequenced.

The amount of starting template can affect the efficiency of LCN-HRM due to the masking effect of wild-type allele, when it is present at too high levels. On the contrary, the use of suboptimal amounts of template increases the rate of non-informative reactions giving no amplification. Using Ct values to estimate the effective number of template molecules allows for the ready choice of an optimal dilution to approximately four copies.

### LCN-HRM for the detection of low level mutations in clinical samples

LCN-HRM was used to investigate the possible cause of discordant results between HRM and sequencing. In our previous HRM study of *EGFR *and *KRAS *mutations in FFPE clinical samples, we observed that the number of HRM positive samples were higher than sequencing positive samples [15]. In this study, we tested selected samples from that study with LCN-HRM to investigate the apparent discrepant results between sequencing and HRM.

One sample, TX450, was selected for LCN-HRM analysis because although it was sequencing negative for a *KRAS *exon 2 mutation, HRM showed similar aberrant melting in replicates indicating the presence of a mutation. The sample had an estimated 20% tumour content at histological review. The detection of the identical *KRAS *mutation (c.34G>T, p.G12C) in 19 multiple independent LCN-HRM replicates proves that the discordant results originated from the different sensitivity of the two methods.

### Frequent occurrence of artefacts in FFPE DNA samples

Another possible cause of discrepancy between HRM and sequencing is due to PCR artefacts as various non-identical sequence variations were identified in the other three FFPE samples tested. Artificial sequence changes are reportedly mediated by DNA polymerase error and/or damaged DNA templates [22,23]. A variety of DNA polymerase-mediated sequence changes can occur during PCR amplification including single base substitutions, deletions and insertions. Among these changes, single base substitutions result from misincorporation of incorrect dNTPs and are the most common type of artefactual change. DNA damage induced during tissue fixation and storage process is another well-documented source of PCR artefacts [23]. These PCR artefacts are randomly distributed and thus are masked by the abundant normal sequences if high amounts of starting template are used. In HRM analysis, a higher frequency of aberrant melting is observed with FFPE DNA compared to high quality DNA sample as the individual random PCR artefacts are cumulatively reflected on the resulting melting curve (results not shown).

### Determining the origin of PCR artefacts

To understand whether the fidelity of DNA polymerase is associated with the generation of PCR artefacts, we used three DNA polymerase kits possessing different levels of proofreading activity. The frequency of LCN-HRM positive reactions was compared in the *EGFR *exon 21 assay using a FFPE sample, TX86, which had previously shown aberrant HRM melting profiles. The rate of PCR artefacts (which were confirmed by sequencing) was not reduced as the fidelity of DNA polymerase was increased (Table 2). Thus, the damaged DNA template is likely to be the principal cause of PCR artefacts. In support of this, when a peripheral blood DNA was tested by LCN-HRM using the same conditions for *KRAS *exon 2, no amplicons showing aberrant melting were detected (data not shown).

It is interesting that a higher LCN-HRM positive rate is seen in LCN-HRM performed with the DNA polymerase kit having the highest proofreading activity. This may be explained by considering that mutation-prone translesional extension at damaged DNA sites occurred more frequently, resulting in subsequent generation of more artefactual changes. The occurrence of translesional synthesis at abasic sites has been previously reported with a Taq polymerase with proofreading activity [24].

It is important to distinguish PCR artefacts from true mutations in the results obtained from FFPE material by LCN-HRM. The detection of an identical nucleotide change at one position in over two or more independent PCR products is supportive of a true mutation whereas nonidentical changes randomly distributed in the template sequence should be considered as PCR artefacts. In addition, weight should be given to those mutations that are observed at well known (canonical) sites of mutation over those mutations that occur at non-canonical sites. Using these criteria, the identical *KRAS *mutation (c.34G>T) detected in multiple independent LCN-HRM products amplified from the TX450 can be considered as a true mutation. However, any variations that fail to be confirmed would be more appropriately interpreted as PCR artefacts as seen in TX13, TX49 and TX86 (Tables 1 and 2, Figure 4).

Fixation of tissue specimens in neutral buffered formalin (10%) is a widely used preservation method as it helps to maintain morphological features [23]. The adverse effect of formalin on DNA quality results in fragmentation of DNA into small sizes [25]. In addition, the incidence of PCR artefacts has increased the risk of misinterpretation of results obtained from formalin-fixed tissues [26,27]. Furthermore, studies of chemical reactions between formaldehyde and DNA have demonstrated several chemical interactions; formation of hydroxymethyl groups and methylene bridges, generation of apurinic and apyrimidinic sites, and hydrolysis of phosphodiester bonds [28,29]. As a result, more frequent DNA polymerase errors have been detected on FFPE material than fresh frozen tissues [26,30].

Although the exact mechanism for the artefacts has not been elucidated, several explanations are possible. Firstly, adenosine residues tend to be incorporated complementary to damaged or cross-linked cytosine nucleotides, due to the so-called "A-rule", generating G to A (or C to T) mutations [26,31]. In this study, nearly all the base changes were G to A or C to T mutations (Tables 1 and 2). In addition, when Taq polymerase encounters damaged templates, it has a propensity to stop nucleotide incorporation and to insert an non-complementary adenosine residue into the strand being synthesised. The formalin-induced DNA damage promotes jumping of the extending primer to another template where extension of the primer continues [32].

As FFPE tissue is frequently used for genetic analysis, the results should thus always be cautiously interpreted. Tsao et al. reported an incidence of 45 *EGFR *mutations in 177 NSCLC patients, comprising 13 deletions and 32 point mutations [33]. However, due to the high frequency (53 percent) of novel variants with predominantly transition changes (92 percent) in this study as well as the lack of adequate confirmation, those changes were suggested to be PCR artefacts [34,35], which is consistent with our observations.

Recently, a large number of non-canonical *EGFR *mutations were reported in both the stromal and epithelial tissue in breast cancer [36]. The same group also reported the occurrence of *TP53 *mutations in both the stromal and epithelial tissue in breast cancer [37]. However, in an independent study, no *TP53 *mutations were detected in stroma of 17 breast cancer or breast cancer-associated fibroblast cultures [38]. The vast majority of the "mutations" reported are thus likely to be the same type of PCR artefacts that we have observed in several of our samples especially as the variants occurred at non-canonical sites and were not adequately confirmed by sequencing of independent PCR products. Consistent with this, the predominant change seen in the samples was a transitional G>A (C>T) change [39,40].

In many cases, mutation analysis of FFPE samples in routine diagnostic situations involves low copy numbers being amplified due to the combination of limited amounts of template and degradation of DNA. Thus, a low copy number may arise inadvertently. Such cases are usually marked by late amplification and often by heterogeneity of melting patterns between samples. Interpretation is similar to that above. Sequencing is necessary to interpret the results and the presence of a mutational change must be confirmed using sequencing of independent reactions. In some cases, further dilution may be warranted to optimise the copy number being analysed.

In conclusion, we have developed a new screening method, LCN-HRM, for detection of low levels of sequence variation. Analysis of multiple replicates of low copy numbers of template allows both the detection of templates with variant sequence (mutations) and enables sequencing to identify the precise changes involved.

The previously reported discrepant results between HRM and sequencing are often due to low level mutant alleles but artefacts due to amplification of low copy numbers or to damaged DNA from FFPE tissues must also be considered. The interpretation of LCN-HRM results should thus be based on the reproducibility and position of detected sequence changes.

## Competing interests

The authors declare that they have no competing interests.

## Authors' contributions

HD participated in the study design, carried out the molecular genetic studies and co-wrote the manuscript. AD conceived the study, participated in the study design and co-wrote the manuscript. Both authors read and approved the final manuscript.
